# The Effect of Poly (Glycerol Sebacate) Incorporation within Hybrid Chitin–Lignin Sol–Gel Nanofibrous Scaffolds

**DOI:** 10.3390/ma11030451

**Published:** 2018-03-19

**Authors:** Tuerdimaimaiti Abudula, Lassaad Gzara, Giovanna Simonetti, Ahmed Alshahrie, Numan Salah, Pierfrancesco Morganti, Angelo Chianese, Afsoon Fallahi, Ali Tamayol, Sidi A. Bencherif, Adnan Memic

**Affiliations:** 1 Center of Nanotechnology, King Abdul Aziz University, Jeddah 21589, Saudi Arabia; 1202908@gmail.com (T.A.); aalshahri@kau.edu.sa (A.A.); nsalah@kau.edu.sa (N.S.); 2 Department of Chemical and Materials Engineering, Faculty of Engineering, King Abdul Aziz University, Jeddah 21589, Saudi Arabia; 3 Center of Excellence in Desalination Technology, King Abdul Aziz University, Jeddah 21589, Saudi Arabia; lassaadgzara@gmail.com; 4 Department of Public Health and Infectious Diseases, “Sapienza” University of Rome, 00185 Rome, Italy; giovanna.simonetti@uniroma1.it; 5 Department Mental/Physical Health and Preventive Medicine, Campania University, L.Vanvitelli, 80121 Naples, Italy; pierfrancesco.morganti@mavicosmetics.it; 6 Nanoscience Centre MAVI, 04011 Aprilia, Italy; 7 Chemical, Materials, Environmental Engineering Department, “Sapienza” University of Rome, 00185 Rome, Italy; angelo.chianese@uniroma1.it; 8 Department of Chemical Engineering, Northeastern University, Boston, MA 02115, USA; afsoonfalahi@gmail.com (A.F.); s.bencherif@northeastern.edu (S.A.B.); 9 Department of Mechanical & Materials Engineering, University of Nebraska, Lincoln, NE 68588, USA; ali.tamayol@gmail.com; 10 Department of Bioengineering, Northeastern University, Boston, MA 02115, USA; 11 Harvard John A. Paulson School of Engineering and Applied Sciences, Harvard University, Cambridge, MA 02138, USA; 12 UMR CNRS 7338 Biomechanics and Bioengineering, University of Technology of Compiègne, Sorbonne University, 60200 Compiègne, France

**Keywords:** electrospinning, hybrid nanofiber, chitin–lignin, sol–gel composite, PGS, mechanical properties

## Abstract

Chitin and lignin primarily accumulate as bio-waste resulting from byproducts of crustacean crusts and plant biomass. Recently, their use has been proposed for diverse and unique bioengineering applications, amongst others. However, their weak mechanical properties need to be improved in order to facilitate their industrial utilization. In this paper, we fabricated hybrid fibers composed of a chitin–lignin (CL)-based sol–gel mixture and elastomeric poly (glycerol sebacate) (PGS) using a standard electrospinning approach. Obtained results showed that PGS could be coherently blended with the sol–gel mixture to form a nanofibrous scaffold exhibiting remarkable mechanical performance and improved antibacterial and antifungal activity. The developed hybrid fibers showed promising potential in advanced biomedical applications such as wound care products. Ultimately, recycling these sustainable biopolymers and other bio-wastes alike could propel a “greener” economy.

## 1. Introduction

Rapid population growth, environmental pollution, and excessive material and energy consumption are driving the development of technologies using renewable “green” resources [1,2]. Among the renewable “green” materials, food and agricultural bio-waste are commonly available all around the world [3]. Approximately 1.3 billion tons of bio-waste are produced each year, accounting for almost one-third of the total food production [3,4]. Recycling and reusing these bio-wastes would not only contribute to economic benefits, but also reduce the negative impact of waste accumulation on the environment, ecosystems, and public health [5,6]. 

Many derivatives of food and agricultural waste exhibit properties like biocompatibility, renewability, and biodegradability, with diverse functionalized chemical components that have many favorable applications [2,5]. Chitin and lignin are excellent examples of bio-waste derived products that could be used in various industries. Chitin is a long chain sugar-like biopolymer consisting of the glucose derivative *N*-acetyl-glucosamine [7]. It is the second most common natural polymer after cellulose, as chitin is the main component of crustacean crusts and cell walls in fungi [5,8,9]. Similarly, lignin, a phenolic biopolymer, is also abundantly available as a byproduct of plant biomass [5,10]. In the past, these polymers were shown to be highly biodegradable, with little or no observable toxicity and were classified as “skin-friendly” [5,6]. In terms of their physical properties, chitin exhibits remarkable water absorption capability and moisturizing efficiency [1]. On the other hand, lignin has various barrier properties against UV radiation and toxic chemicals [11]. Antimicrobial performance is also another attractive feature of these materials, in which chitin is highly active in inhibiting the growth of many gram-negative bacteria [12], and lignin has antimicrobial activity against gram-positive bacteria and fungi [13]. In addition, when combining positively charged chitin and negatively charged lignin, novel composites able to adsorb and release various ingredients could be fabricated [5].

Several research groups have centered their research on combining biodegradable polymers originating from organic bio-waste. For example, Morganti et al. developed chitin–lignin (CL)-based sol–gel composites by using a nontoxic, non-immunogenic, water soluble, strongly polar polymer composite incorporating polyethylene oxide (PEO) [11,14] as a carrier polymer, and created advanced nonwoven scaffolds [4,5]. Potential applications of these scaffolds were found to include (amongst others) advanced medical devices, wound healing, and air and water purification [5]. However, adequate mechanical properties of these scaffolds are still lacking and require improvement for large-scale industrial and biomedical applications. One way to enhance the mechanical properties of these naturally-derived polymers is by developing hybrid systems with synthetic polymers [15,16,17]. 

Poly(glycerol sebacate) (PGS) is a recently developed synthetic elastomer first reported by Wang et al. in 2002 [18], which can be synthesized by polycondensation of equimolar ratios of glycerol and sebacic acid [18,19]. Polymer synthesis is simple and cost-effective [20], and both reagents have been approved by Food and Drug Administration [21] which could propel its biomedical applications. For example, this material was extensively studied for many biomedical applications, including tissue engineering [19,22], drug delivery [23,24], wound healing [25], and implantable devices [26] due to its excellent elastomeric and ductile mechanical properties, erodible surface, controllable degradation profile, and non-toxic characteristics [19,20,21]. 

There are many methods by which hybrid natural–synthetic polymeric composite scaffolds can be fabricated. One method that has been used extensively to produce fibrous scaffolds is electrospinning [16,19,27]. In this method, the formation of ultrathin fibers is achieved by applying high voltage electric field on the viscoelastic solution [16]. It is a simple and flexible technique to fabricate both natural and synthetic polymer-based scaffolds that are meant to mimic the extracellular matrix (ECM). The scaffolds often have a highly porous structure that can be made with tunable mechanical, surface, and biochemical properties [16,19,27]. Furthermore, hybrid scaffolds can easily be fabricated by electrospinning of polymer blends. However, several challenges can exist including matching solvents, low molecular weight, low melting points, and low viscosity for some polymers [28]. Generally, pure PGS is difficult to electrospin on its own, however, when blended with other readily electrospun materials, uniform structures without fiber fusion or beads have been reported [19,28]. 

In the current study, we synthesized a hybrid nanofibrous scaffold that combined a CL sol–gel mixture with PGS by standard electrospinning. Chemical structures, morphology, and mechanical and thermal characteristics, as well as the antimicrobial properties of these hybrid fibers were examined in order to design the best electrospun composite. We assessed the effect of PGS in its ability to create a hybrid scaffold that yields uniform fiber structure, excellent thermal and mechanical performance, and improved antimicrobial activity. 

## 2. Materials and Methods 

### 2.1. Materials

The following chemicals were used to prepare hybrid electrospun fibers from the CL sol–gel composite and PGS: chitin nanofibrils in the form of 2% water suspension (MAVI, Sud Srl, Aprilia, LT, Italy); bio-lignin (CIMV, Neuilly-sur-Seine, France); polyethylene oxide (PEO) (Amerchol, DowCorning, Milano, Italy); glycerol (Sigma-Aldrich, St. Louis, MO, USA); sebacic acid (Sigma-Aldrich, St. Louis, MO, USA); ethanol (Sigma-Aldrich, St. Louis, MO, USA); and deionized water. Synthesis of PGS was achieved by polycondensation of glycerol and sebacic acid in a 1:1 ratio, according to a previously reported procedure [16].

### 2.2. Preparation of Solutions

All the components including water were measured by weight base, therefore 30 wt % of chitin nanofibrils, 0.1 wt % of bio-lignin, and 7 wt % of PEO were successively added into 62.9 wt % deionized water to produce the sol–gel mixture, and pH of the mixture was adjusted to 10.5 by slowly adding 0.1 M of NaOH. Then, the mixture was covered and stirred for 48 h to obtain a homogeneous CL sol–gel solution.

PGS solution (25 w/v) was obtained by dissolving PGS in ethanol with magnetic stirring for 30 minutes. Then sol–gel and PGS solutions were mixed together in volume ratios of 100/0, 99/1, 95/5, 90/10, 85/15, 80/20, 70/30, and 50/50, and stirred for another 30 minutes to achieve coherent dispersion of the materials with the solution. 

### 2.3. Electrospinning

Electrospinning was performed in a Nanon 101A electrospinning setup (NANON Supply, MECC, Fukuoka, Japan). A 5-mL syringe (Becton-Dickinson, Franklin Lakes, NJ, USA) and an 18-gauge blunt metallic needle (NANON Supply, MECC, Fukuoka, Japan) were connected to approximately 10 cm of Teflon tube (Cole-Parmer Instrument Company, Vernon Hills, IL, USA) to deliver the solution with a feed rate of 0.3 mL/h. Then, 18 kV of voltage over 150 mm of distance was applied to stretch the solution, and the produced fibers were collected on flat aluminum sheet. The collected samples were kept for two days at room temperature for natural drying before performing any characterization test. 

### 2.4. Characterization

Morphological properties of the electrospun hybrid fibers were examined by field emission scanning electron microscopy (FESEM, JEOL JSM 7600F, Tokyo, Japan). Size distribution of the fibers was calculated by image processing in “Image J” software (1.50i, National Institutes of Health, Bethesda, MD, USA) [27]. The SEM micrograph of the sample was passed through adjustment, smoothing, thresholding, and noise removal. Then, fiber sizes were calculated by Euclidean distance transform. 

Fourier transform infrared (FTIR) spectra of the samples were obtained using the Thermo Scientific ATR (Attenuated total reflection)-FTIR spectrometer (Thermo Fisher Scientific, Waltham, MA, USA) in a wavelength range of 400~4000 cm^−1^ with 0.5 cm^−1^.

Thermal properties of the samples were analyzed by differential scanning calorimetry (DSC) using MICRO DSC3 EVO (Setaram Inc., Hillsborough Township, NJ, USA). The samples loaded on a low-volume aluminum crucible (S08/HBB3740) were first heated up to 180 °C at 5 °C/min, and were then cooled down to 25 °C at 3 °C/min. Nitrogen was swept over the sample with 10 ml/min of feed rate during the experiment. 

### 2.5. Mechanical Test

Mechanical properties of the hybrid fibers were evaluated by performing uniaxial tensile test measurement in the universal tensile machine (Lloyd Instruments Ltd., Bognor Regis, UK). The prepared electrospun fiber sheet was cut in rectangular shape with dimension of 40 mm × 10 mm and the sheet thickness was measured by electronic caliper (0.1–0.5 mm); then it was stretched with a cross-head speed of 10 mm/min. The load elongation curve of the sample was recorded by NEXYGENPlus Materials Testing Software(Plus 3.0, Lloyd Instruments Ltd., Bognor Regis, UK), and each sample was tested at least three times to calculate average Young’s modulus, tensile strength, and strain at failure.

### 2.6. Antibacterial and Antifungal Activity Test

Diffusion tests were performed according to the Standard SNV 195920-1992 to evaluate the antimicrobial activity of the samples as previously described [29]. These consist of a diffusion test in agar: the electrospun fibers, after having cut them to the same size, were placed in contact with bacterial strains *Staphylococcus aureus* (ATCC 6535), or *Escherichia coli* (ATCC 8734), and fungi *Candida albicans* (ATCC 10231) on agar plates. Samples treated were incubated for 24 h at 37 °C. After the incubation time, the inhibition zone around and beneath the samples was evaluated, and the activity of the samples was defined according to the antibacterial degrees provided by the standard. When an inhibition zone is observed both beneath and surrounding the loaded polymers, the antibacterial properties are defined as “good”. When growth inhibition area is observed under the sample only, the antibacterial properties are defined as “sufficient.” If the sample is totally covered by the bacteria as well as the area under the loaded polymers, the antibacterial properties are defined as “insufficient”.

## 3. Results

**Figure 1:**
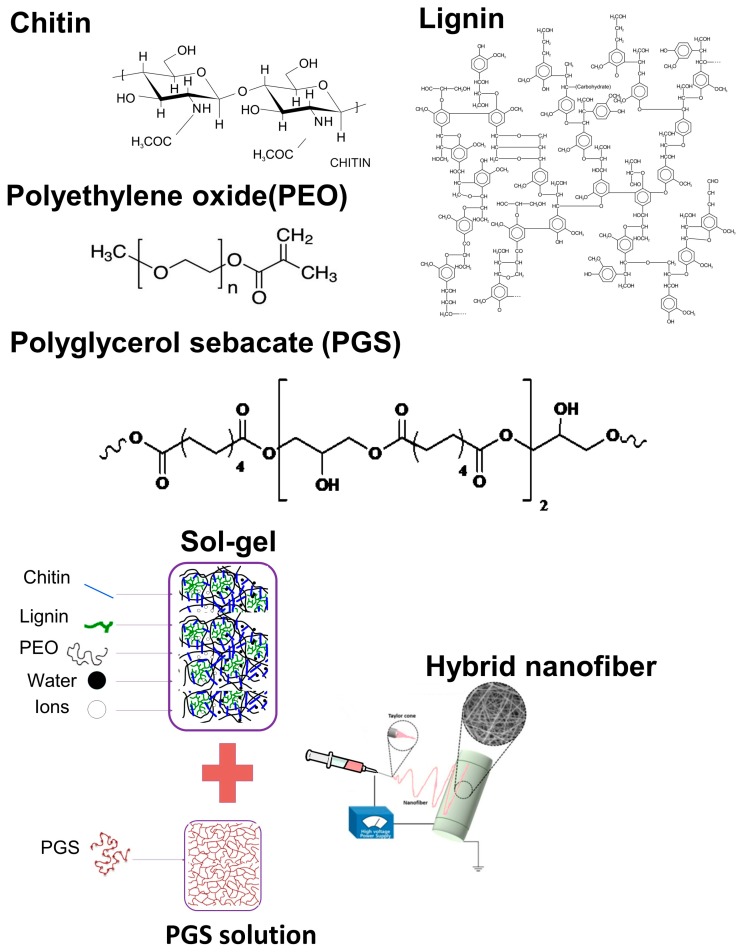
Systematic representation of hybrid fiber synthesis from the chitin–lignin (CL) sol–gel composite and PGS by standard electrospinning.

Schematic representation of hybrid fibers synthesized from CL sol–gel and PGS by electrospinning is given in Figure 1. The first step was to optimize the electrospinning fabrication process for the CL sol–gel that could then be hybridized with PGS. The electrospinning parameters that required tuning included applied voltage, feed rate, and nozzle-collector distance. We wanted to ensure that CL sol–gel alone would yield smooth and uniform fibers before adding PGS. Most suitable electrospinning conditions to produce CL based sol–gel fibers had 0.3 mL/h of feed rate, 18 kV voltage, and 15 cm of needle-collector distance. PGS incorporation into CL sol–gel was accomplished by PGS dissolution in ethanol since it is a water-miscible solvent that could be combined with the CL sol–gel counterpart. 

**Figure 2:**
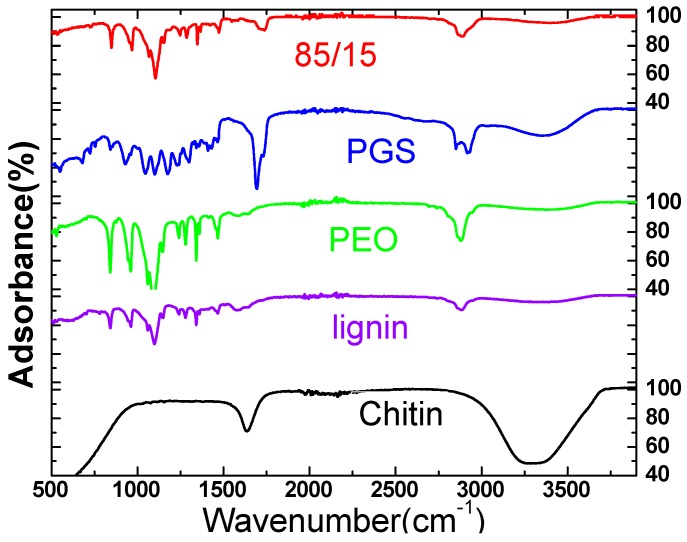
FTIR spectra of the hybrid fiber with 15 vol % of PGS solution (85/15) and its individual components.

FTIR spectra of the hybrid fibers with 15 vol % of PGS solution are presented in Figure 2, in addition to the spectra for the individual components. The spectra depict FTIR fingerprints for all the individual components within the hybrid fibers. The most dominant peaks appearing at 1141 cm^−1^ and 1098 cm^−1^ (corresponding to the C-O-C bond), and 1060 cm^−1^, 961 cm^−1^, and 841 cm^−1^ (corresponding to the =C-H bond) predominantly exist in PEO, PGS, and lignin. Similarly, the peaks at 1466 cm^−1^, 1359 cm^−1^, and 1341 cm^−1^ (corresponding to the -C-H bond) could also be derived from PEO, PGS, and lignin. In addition, the peak observed at 2881 cm^−1^ (corresponding to the –O-H bond) shows the presence of PGS, and the peaks at 403 cm^−1^ and 431 cm^−1^ (corresponding to the -N-H bond) show the presence of chitin in the hybrid fiber. We noticed that no significant shifts of the IR (Infrared) bands were observed for the hybrid fiber compared to its individual component. This suggests that the chemical compositions of the individual components remained the same without chemical reactions or new covalent bond formation during the fiber fabrication process.

**Figure 3:**
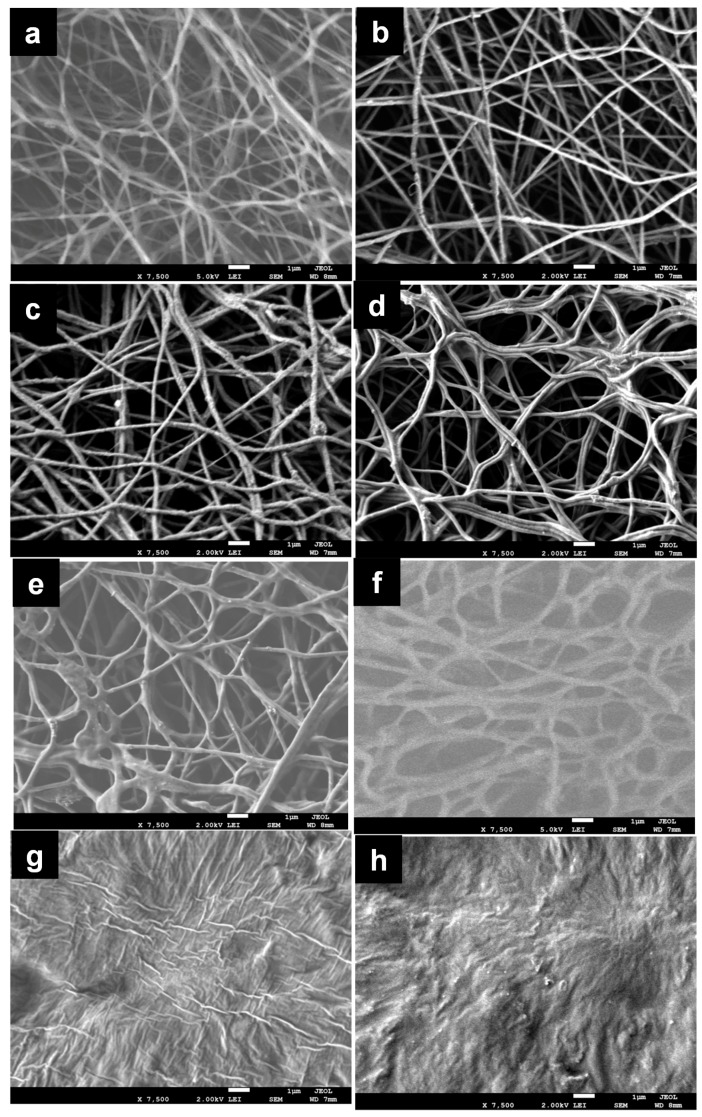
SEM micrograph of pure sol–gel fiber (**a**) and hybrid fibers in which the volume ratios of CL sol–gel solution and PGS solution were 99/1 (**b**); 95/5 (**c**), 90/10 (**d**); 85/15 (**e**); 80/20 (**f**); 70/30 (**g**); and 50/50 (**h**).

**Figure 4:**
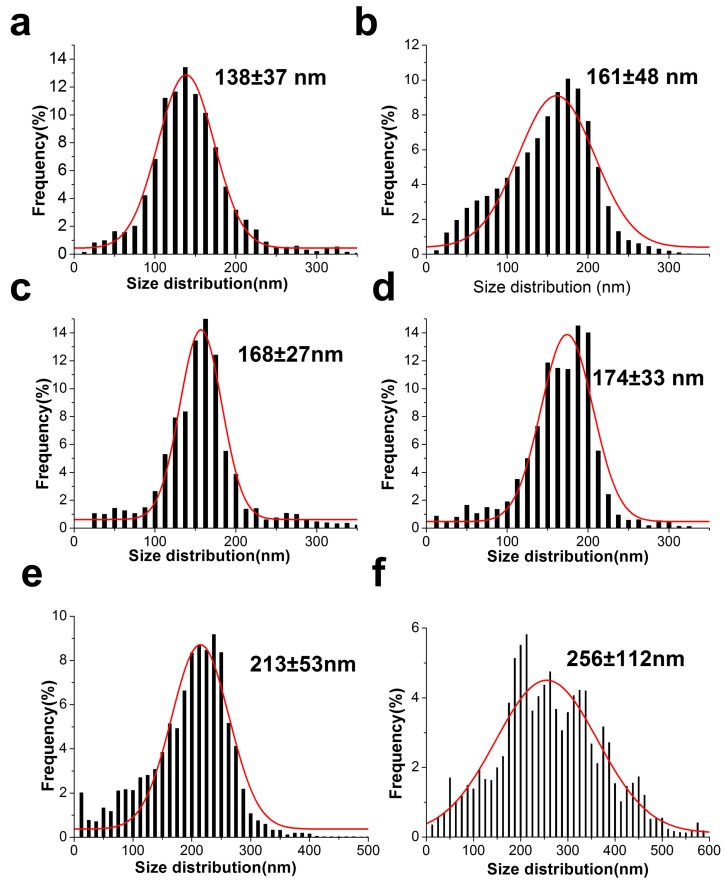
Size distribution and average fiber diameter sizes of pure sol–gel fiber (**a**) and hybrid fibers in which the volume ratios of CL sol–gel solution and PGS solution were 99/1 (**b**); 95/5 (**c**); 90/10 (**d**); 85/15 (**e**) and 80/20 (**f**).

Next, Figure 3 depicts morphological characterization of electrospun CL fibers and hybrid sol–gel fibers and PGS at various volume ratios. The figure implies that PGS content significantly affects the formation and morphology of the resulting hybrid fibers. Smooth and uniform fibers were observed from the SEM images (Figure 3a–d) with low content of PGS (0–10 vol %), which indicates homogenous incorporation of PGS along with the CL fibers. With increasing volume percentage of PGS solution to above 10%, fibers appear to gradually delaminate during electrospinning (Figure 3e–f) yielding multiple fiber populations. This could be attributed to PGS forming both separate individual fibers and creating distorted fiber fusions, perhaps due to its low viscosity and low glass transition temperature [28,30]. Fiber fusion became more pronounced by further increasing PGS contents, and no noticeable fibers were found at 30 vol % and 50 vol % of PGS. Overall, it was possible to obtain electrospun hybrid fibers from the blends of sol–gel and PGS when volume percentage of PGS solution remained below 20 vol %. Diameter distributions of electrospun hybrid fibers prepared using different ratios of PGS are presented in Figure 4. Diameter of pure sol–gel fiber was found to be 138 ± 37 nm. This is comparable to previously reported needleless electrospinning using 45–75 kV of voltage over a 10–16 cm distance, which generated a minimum average fiber diameter of 138 nm [4]. The current results show that similar fiber diameter can be obtained by “standard” nozzle-based electrospinning, but at a significantly lower voltage. When PGS was incorporated, fiber diameters were slightly increased; while mostly uniform and smooth fibers were observed up to 15 vol % of PGS in the pre-spinning solution. Further increasing PGS content caused drastic changes, broader variation of fiber diameters, and increases in fiber size. This is perhaps due to formation of thicker fibers combined by several thin fusion fibers as the result of PGS incorporation or due to solution viscosity as mentioned before.

**Figure 5:**
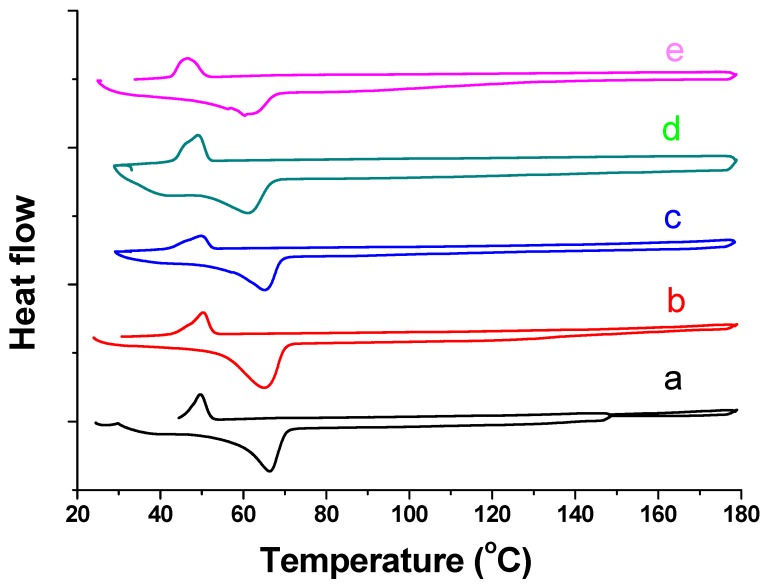
Differential scanning calorimetry (DSC) curve of pure sol–gel fiber (a) and hybrid fibers in which volume ratios of CL sol–gel solution and PGS solution were 95/5 (b); 90/10 (c); 85/15 (d) and 80/20 (e).

Figure 5 illustrates the DSC curves for hybrid fibers combining both heating and cooling process in the temperature range of 25 to 180 °C. In the heating process, the pure sol–gel fiber showed a strong and sharp endothermal peak at 66.4 °C corresponding to the melting point of PEO [31,32] and an embossed endothermal change between 30 and 150 °C which could be the result of evaporation of binding moisture or the so-called “gel” in the CL sol–gel composite [33]. In the cooling process, the sol–gel fiber exhibited only one sharp exothermic peak at 49.7 °C attributed to the crystallization temperature of PEO [34]. DCS curves for hybrid fibers with different PGS content were similar to those of the pure sol–gel fibers. Only the melting temperature and the crystallization temperature were found to be lowered in proportion to the increasing PGS content. It was reported in the literature that PGS has a melting temperature of 9.6 °C and crystallization temperature of −18.8 °C [35]. Therefore, the results indicate that PGS is miscible with the sol–gel composite, and all the various polymers are dispersed coherently in the hybrid fiber [36]. 

**Figure 6:**
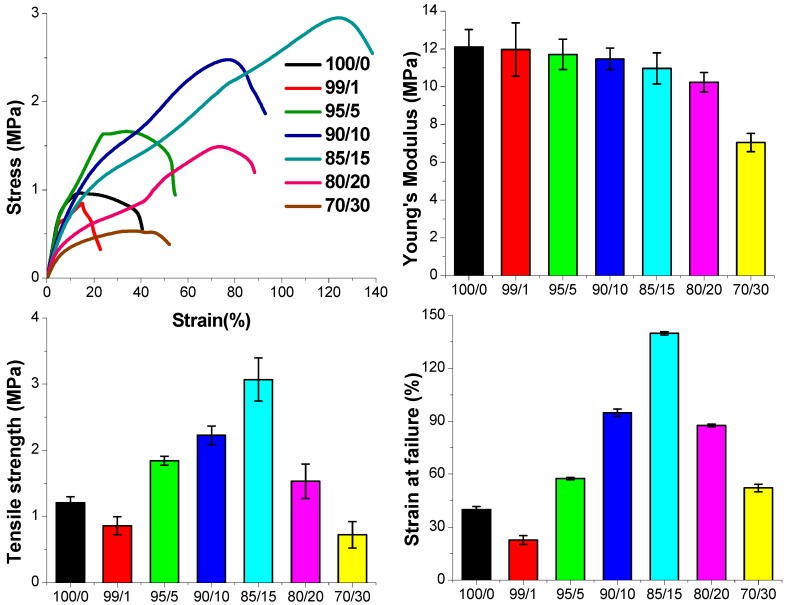
The strain–stress curves and corresponding Young’s modulus, tensile strength and elongation at failure values of pure sol–gel fiber (100/0) and hybrid fibers with different volume ratios of CL sol–gel solution and PGS solution.

The stress–strain curves, and corresponding tensile modulus, tensile strength, and elongation at failure values of the electrospun hybrid fibers are given in Figure 6. For pure CL sol–gel fibers, Young’s modulus, tensile strength, and elongation at failure values were found to be 14.8 ± 0.9 MPa, 1.2 ± 0.1 MPa, and 40 ± 2%, respectively. By incorporating PGS, mechanical properties of the sol–gel fibers can be greatly improved, especially tensile strength and stretchability. When PGS was added to the sol–gel mixture, tensile strength, and elongation at failure gradually improved with increasing volume percentage of PGS (up to 15 vol %). The hybrid fiber with 15 vol % of PGS solution exhibited at 3.1 ± 0.3 MPa the highest tensile strength and at 140 ± 1% the longest strain, which is almost triple that of pure sol–gel fibers. This could be attributed to strong interactions between CL sol–gel and PGS polymers during fiber fabrication. Also, the compliance and stretchability of the hybrid fiber were proportional to PGS content, as previously reported in the literature [27,37]. Further increasing the PGS content to 20 vol % or higher reduced the ultimate strength and elongation of the hybrid fibers. At this point, there might be a higher phase separation of the two materials leading to weak interaction between the individual components. Tensile moduli of the hybrid fibers slightly decreased by increasing the PGS content, which was associated with a softening effect of PGS [28,38]. 

**Figure 7:**
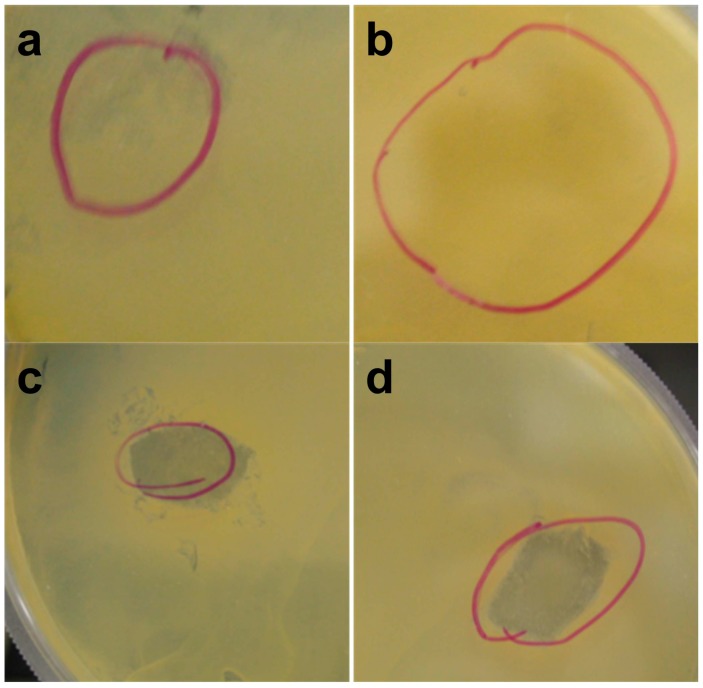
Growth inhibition of *Staphylococcus aureus* ATCC 6535 by the sol–gel fiber (**a**); and the hybrid fibers in which the volume percentage of PGS solution is 10% (**b**); 15% (**c**); and 20% (**d**). The red circles define the original locations of the samples.

Antibacterial and antifungal tests were performed by incubation at 37 °C for 24 h on loaded polymers as listed in Table 1. Although antibacterial activity of chitin and lignin was previously reported on by several researchers [4,13,39], pure sol–gel fiber and hybrid fiber with less than 10 vol % of PGS solution did not show any significant inhibition effect on bacterial and fungal growth. Although the exact mechanism is unknown, this might be explained by immediate solubility of these fibers in the aqueous media, which causes destruction of long molecular chains of the chitin and lignin, and reduction in the molecular weight [40]. Antibacterial and antifungal activity decreased due to the short surface interaction between microorganisms and the fibers as they quickly dissolve [40,41]. The hybrid fibers with 15 vol % or higher PGS interestingly showed inhibition across the two bacteria tested. A clear zone of inhibition was observed both surrounding and beneath the hybrid fibers against *S. aureus* and *E. coli* strains. Figure 7 specifically describes the presence and the shape of a clearly visible microbial-free area for the hybrid fibers with 15 and 20 vol % of PGS against *S. aureus.* The retarding effect of PGS on the solubility of the sol–gel fiber might be the main factor for the improved antibacterial performance of these hybrid fibers. Additionally, glycol acid, which is an acidic byproduct of PGS during degradation, could also promote antibacterial activity [42]. An effective antifungal performance, however, was not observed except the sample with 30 vol % of PGS, which might be attributed to a lower antifungal activity of individual components in the hybrid fibers [13]. Similarity in chemical composition between chitin and the fungi wall could also be another reason for the inferior antifungal performance of the pure and hybrid fibrous scaffolds [43].

## 4. Discussion

Hybrid nanofibers composed of chitin–lignin composite and PGS were successfully developed by the standard electrospinning technique. FTIR results confirmed the incorporation of all components within the fiber without any observable chemical interaction. SEM data suggested that volumetric percentage of PGS solution should be less than 20% to obtain a smooth and uniform fiber-like structure. DSC results show that addition of PGS only trivially affects the thermal behavior of the sol–gel composite. Mechanical properties of hybrid fibers were found to be tuned by both PGS content and fiber structure, and the best mechanical performance was obtained for the hybrid fiber in which the volume percentage of PGS solution was 85/15. The highest amount of PGS was found to impart the sol–gel based hybrid fibers with the best antimicrobial properties, with activity both against bacteria and fungi. On the contrary, by decreasing the concentration of PGS, the fibers did not show antifungal activity. Activity against bacteria and fungi is a fundamental requirement for possible broad-spectrum antimicrobial scaffolds for applications in wound healing for example. Overall, the mechanical and antibacterial performance of CL based sol–gel fibers was greatly improved by incorporating PGS, and an ECM-like fibrous structure was shown. Therefore, this bio-waste based hybrid fiber could be used in a much wider range of applications in biomedical fields (for example in wound dressing), and its use would decrease the demands on limited fossil fuel resources and minimize the negative impacts of waste accumulation.

## Figures and Tables

**Table 1:** Antimicrobial activity of loaded polymers against *Staphylococcus aureus* ATCC 6535, *Escherichia coli* ATCC 8734 and *Candida albicans* ATCC 10231.

Loaded Polymers	*Staphylococcus aureus* ATCC 6535	*Escherichia coli* ATCC 8734	*Candida albicans* ATCC 10231
70/30	Good	Good	Good
80/20	Good	Good–Sufficient	Not sufficient
85/15	Good	Good–Sufficient	Sufficient–Not sufficient
90/10	Sufficient–Not sufficient	Sufficient–Not sufficient	Not sufficient
95/5	Sufficient–Not sufficient	Sufficient–Not sufficient	Not sufficient
99/1	Not sufficient	Not sufficient	Not sufficient
100/0	Not sufficient	Not sufficient	Not sufficient

The activity was defined according to the antibacterial degrees provided by Standard SNV195920. Good: inhibition zone in both close and under the loaded polymers; Sufficient: growth inhibition area under the sample only; Not sufficient: no growth inhibition. The results are reported in the table for a duplicate experiment (n = 2).
